# Urinary Concentrations of Diisoheptyl Phthalate Biomarkers in Convenience Samples of U.S. Adults in 2000 and 2018–2019

**DOI:** 10.3390/toxics7040053

**Published:** 2019-10-11

**Authors:** Manori J. Silva, Lee-Yang Wong, James L. Preau, Ella Samandar, Emmanuela Obi, Antonia M. Calafat, Julianne C. Botelho

**Affiliations:** Division of Laboratory Sciences, National Center for Environmental Health, Centers for Disease Control and Prevention, Atlanta, GA 30341, USA; lyw8@cdc.gov (L.-Y.W.); nzp4@cdc.gov (J.L.P.); evs9@cdc.gov (E.S.); osn8@cdc.gov (E.O.); aic7@cdc.gov (A.M.C.); gur5@cdc.gov (J.C.B.)

**Keywords:** diisoheptyl phthalate, DiHpP plasticizers, exposure, oxidative metabolites

## Abstract

We know little about the potential health risks from exposure to diisoheptyl phthalate (DiHpP), a plasticizer used in commercial applications. The production of DiHpP ended in the United States in 2010, but DiHpP may still be present in phthalate diester mixtures. To investigate human exposure to DiHpP, we used three oxidative metabolites of DiHpP: Monohydroxyheptyl phthalate (MHHpP), mono-oxoheptylphthalate (MOHpP), and monocarboxyhexyl phthalate (MCHxP) as exposure biomarkers. We analyzed urine collected anonymously in 2000 (*N* = 144) and 2018–2019 (*N* = 205) from convenience groups of U.S. adults using high-performance liquid chromatography coupled with isotope-dilution high-resolution mass spectrometry. We detected MCHxP in all the samples tested in 2000 (GM = 2.01 ng/mL) and 2018–2019 (GM = 1.31 ng/mL). MHHpP was also detected in 100% of the 2018–2019 samples (GM = 0.59 ng/mL) and 96% of the 2000 urine samples analyzed (GM = 0.38 ng/mL). MOHpP was detected in 57% (2018–2019, GM = 0.03 ng/mL) and 92% (2000, GM = 0.19 ng/mL) of samples. The presence of MHHpP, MOHpP, and MCHxP in the 2018–2019 samples suggests recent exposure to DiHpP. Intercorrelations between metabolite concentrations were more significant in samples collected in 2000 than in samples collected in 2018–2019. The differences in urinary metabolite profiles and intercorrelations from samples collected during 2000 and 2018–2019 likely reflects changes in the composition of commercial DiHpP formulations before and after 2010.

## 1. Introduction

Diisoheptyl phthalate (DiHpP), an isomeric mixture of phthalates with branched and linear seven carbon backbones, is used commercially as a plasticizer in vinyl resins. DiHpP can be found in automotive, wire, cable, imitation leather, and flooring products [1]. DiHpP production ended in the European Union and United States in 2010 [2]. However, DiHpP may still be present in phthalate diester mixtures [1] that can be used in consumer products in the United States and European Union. As a result, human exposure to DiHpP can occur.

Animal studies suggest potential adverse health effects from exposure to DiHpP [2,3,4,5,6,7]. In a 28-day repeated oral DiHpP dose toxicity test in male and female F344 rats, body weight gain was inhibited, but liver and kidney weights increased [8]. In a developmental toxicity study, female rats given DiHpP by oral gavage on gestational days 6–20 had increased resorptions and reduced fetal weight [7]. Metabolites of phthalates are often used as biomarkers of exposure [9]. To date, no human data on exposure to DiHpP have been reported. Hence, DiHpP biomonitoring to assess exposure in humans might prove to be valuable.

Oxidative metabolites form during metabolism of phthalates and are excreted in urine [10,11]. Population-based biomonitoring studies use oxidative metabolites of phthalates as biomarkers of exposure [9,12,13,14,15]. Monohydroxyheptyl phthalate (MHHpP) and monocarboxyhexyl phthalate (MCHxP), which formed as DiHpP metabolites, were identified in urine of rats dosed with DiHpP [16]. In our study, we quantified three oxidative metabolites of DiHpP to better understand human exposure to DiHpP.

## 2. Materials and Methods

### 2.1. Chemicals and Equipment

**Figure 1:**
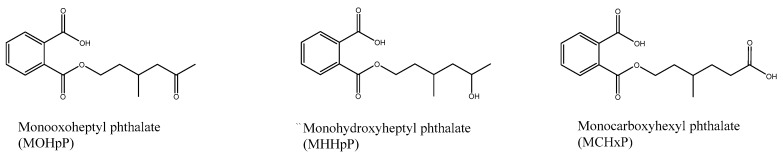
Metabolites used to assess exposure to diisoheptyl phthalate. Only one isomer for each metabolite is shown.

We bought MHHpP, mono-oxoheptyl phthalate (MOHpP), MCHxP (Figure 1), d_4_-MHHpP, d_4_-MOHpP, and d_4_-MCHxP from ADM Germany (>95%). We bought high-performance liquid chromatography (HPLC) grade acetonitrile, water, and methanol (99.8%) from Honeywell Burdick & Jackson (Muskegon, MI, USA). β-glucuronidase (*Escherichia coli*-K12) was purchased from Roche Biomedical (Mannheim, Germany). All chemicals and reagents were used without further purification.

We used a QExactive plus Hybrid Quadrupole-Orbitrap mass spectrometer and Ultimate 3000 high-performance chromatography system (Thermo Fisher Scientific, Waltham, MA, USA) for sample analysis and Xcalibur 2.2 (Thermo Fisher Scientific) for data processing.

### 2.2. Subjects

We collected urine anonymously from demographically diverse groups of U.S. male and female adults from Atlanta, GA, during 2000 and 2018–2019 to study exposure biomarkers to environmental chemicals. No personal information from the subjects was available. Samples were collected between 8:00 a.m. and 5:00 p.m. and were not necessarily first-morning voids. Same donors may have contributed urine in different collection years, during different days, or at different times of day. After collection, samples were stored at −70 °C until analysis. The Centers for Disease Control and Prevention (CDC) Institutional Review Board approved the urine collection and analysis. A waiver of informed consent was requested under 45 CFR 46.116(d) general requirements for informed consent.

### 2.3. Analytical Method

**Figure 2:**
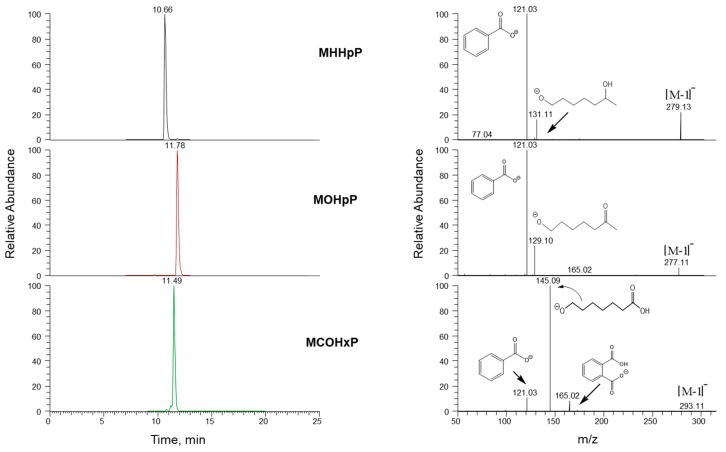
Chromatographic separation and mass spectra of diisoheptyl phthalate metabolites.

We obtained the mass spectra for all three metabolites using analytical standards prepared in acetonitrile and optimized the fragmentation of the precursor ion for each metabolite (Table 1). We adapted published analytical methods for measuring phthalate oxidative metabolites in urine [17,18]. Briefly, urine (0.1 mL) and calibration standards (0.013–130 ng/mL) were spiked with an internal standard solution (5–10 ng/mL) containing d_4_-MHHpP, d_4_-MOHpP, d_4_-MCHxP, and a buffered solution of β-glucuronidase (*Escherichia coli*-K12; 25 μL, pH 6.5, 1 M ammonium acetate). The mixture was incubated at 37 °C for a minimum of 120 min [17,18]. The target analytes in the spiked urine were extracted using on-line solid-phase extraction (Chromolith HighResolution RP-18, 4.6 mm Guard Cartridge; Merck KGaA, Darmstadt, Germany) and chromatographically resolved using high-performance liquid chromatography (Thermo Scientific Betasil phenyl, 3 µm, 150 mm × 2.1 mm column) (Figure 2). We then used high-resolution mass spectrometry in negative ion parallel reaction mode on a QExactive high-resolution mass spectrometer for quantification of the analytes (Table 1). We used the lowest calibration level (0.013 ng/mL) as the limit of detection (LOD).

We also compared the urinary MHHpP concentrations to the hydroxylated metabolites of dibutyl phthalate, diisobutyl phthalate, and di-2-ethylhexyl phthalate concentrations in human urine reported in the 2015–2016 National Health and Nutrition Examination Survey [19].

We used SAS (version 9.4; SAS Institute Inc., Cary, NC, USA) to perform statistical analyses. For metabolite concentrations below the LOD, we imputed a value equal to the LOD divided by the square root of 2 [20]. Statistical significance was set at Pearson correlation coefficient (*p*) < 0.05.

## 3. Results and Discussion

DiHpP has been used in vinyl plastics, including flooring, but no data are available on human exposure. In this proof-of-concept study, we report the urinary concentrations of three oxidative metabolites of DiHpP (MHHpP, MOHpP, and MCHxP). The urine was collected during 2000 and 2018–2019 from convenience samples of U.S. adults not known to be occupationally exposed. We used high-resolution mass spectrometry to resolve analytes from isobaric interferences, which allowed us to quantify the three metabolites at sub-parts per billion concentrations. Because DiHpP consists of multiple isomers with similar physical and chemical properties, metabolites were eluted as broad HPLC peaks with similar mass spectrometric transitions, as reported previously for other isomeric phthalate mixtures [21,22].

Table 2 lists geometric means (GM), select percentile concentrations, and detection frequencies of DiHpP metabolites in urine. We detected MCHxP in all the urine samples tested. MHHpP was also detected in the 2018–2019 (100%) and 2000 (96%) samples. MOHpP was detected less frequently, at 57% in the 2018–2019 samples and 92% in the 2000 samples. The frequent detection of these metabolites in the 2018–2019 samples suggests recent exposure to DiHpP.

The highest concentrations were for MCHxP, followed by MHHpP and MOHpP. However, MCHxP is not a DiHpP-specific metabolite. MCHxP can also be produced by other high-molecular-weight phthalates [23], whereas MHHpP and MOHpP are specific biomarkers for DiHpP. The GM (95% CI) concentrations of MOHpP (0.19 (0.16, 0.24) vs. 0.03 (0.03, 0.04) ng/mL for 2000 and 2018–2019, respectively) and MCHxP (2.0 (1.66, 2.43) vs. 1.31 (1.15, 1.50) ng/mL for 2000 and 2018–2019, respectively) were higher in samples collected in 2000 than in 2018–2019 (Table 2). In contrast, the GM concentrations of MHHpP were higher in samples collected in 2018–2019 (0.59 (0.50, 0.70) ng/mL) than in 2000 (0.38 (0.31, 0.46) ng/mL), perhaps because of differences in the formulations before and after manufacturing changes for DiHpP in the United States. Although, the production of DiHpP was discontinued in 2010 [1], the use of DiHpP in C_6_–C_8_, C_7_–C_9_, and C_6_–C_10_ and other commercial phthalate mixtures [1] might have contributed to exposure in later years.

**Figure 3:**
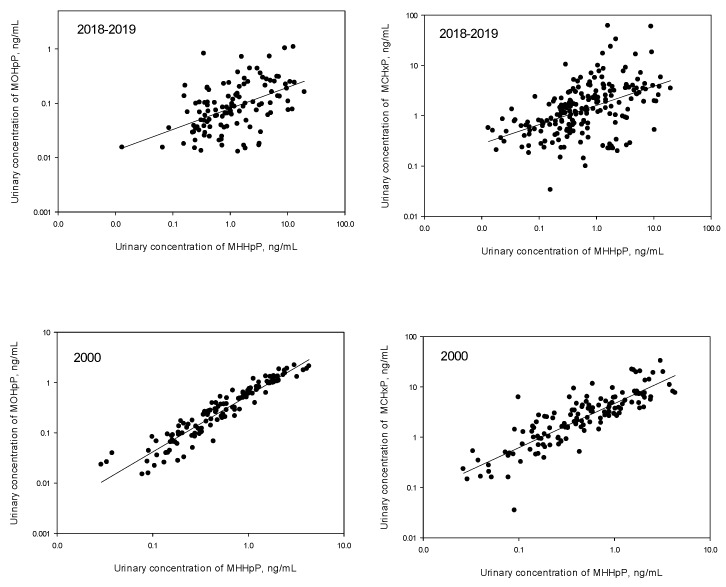
Correlation analyses of urinary concentrations of the metabolites of diisoheptyl phthalate (only the concentrations above the limits of detection are shown).

As expected, the correlation analysis showed statistically significant correlations (*p* < 0.001) between the log_10_-transformed concentrations of MHHpP and MOHpP (Figure 3). The correlation was more significant in samples collected in 2000 (correlation coefficient (r), (95% CI) = 0.94 (0.91, 0.95)) than in samples collected in 2018–2019 (*r*, (95% CI) = 0.59 (0.49, 0.67)) (Figure 3). To explain this finding, we hypothesize that phthalate formulations used before the 2010 ending of DiHpP production in the USA may have included a larger percentage of DiHpP isomers with straight-chain C-backbone, where oxidation to form MOHpP can readily occur. Urinary concentrations of MHHpP also correlated with those of MCHxP (*r*, (95% CI) = 0.87 (0.83, 0.91), 0.49 (0.38, 0.59) for 2000 and 2018–2019, respectively (*p* < 0.001)) (Figure 3), suggesting DiHpP as the primary source for MCHxP in these samples.

**Figure 4:**
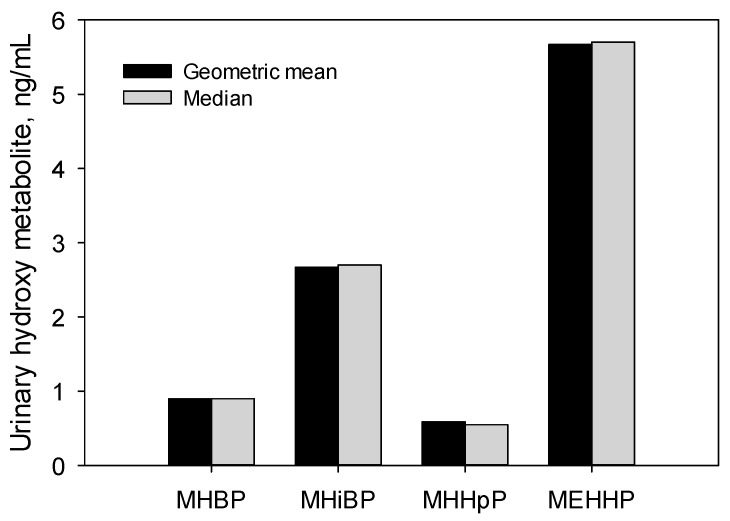
Geometric mean and median concentrations of the hydroxy metabolites of dibutyl phthalate (DBP), diisobutyl phthalate (DiBP), and di-2-ethylhexyl phthalate (DEHP) in urine samples from the 2015–2016 National Health and Nutrition Examination Survey (NHANES) compared with MHHpP (2018–2019 current study). Monohydroxybutyl phthalate (MHBP)-metabolite of DBP; monohydroxy-isobutyl phthalate (MHiBP)-metabolite of DiBP; mono-2-ethyl-5-hydroxyhexyl phthalate (MEHHP)-metabolite of DEHP.

The detection of DiHpP metabolites among a diverse group of U.S. adults suggests exposure to DiHpP in the United States. However, the concentrations of the metabolites were lower than most other phthalate metabolites detected in human urine reported from the 2015–2016 National Health and Nutrition Examination Survey (Figure 4) [19]. These pilot data suggest that the DiHpP metabolites (MHHpP, MOHpP, and MCHxP) can serve as biomarkers of exposure to DiHpP in large-scale biomonitoring studies.

## 4. Disclaimer

The use of trade names is for identification purposes only and does not constitute endorsement by the U.S. Department of Health and Human Services or the Centers for Disease Control and Prevention. The findings and conclusions in this report are those of the authors and do not necessarily represent the views of the Centers for Disease Control and Prevention.

## Figures and Tables

**Table 1:** Analytical parameters for the quantification of diisoheptyl phthalate metabolites.

Parent Chemical	Urinary Metabolite	Internal Standard	MS/MS Scan (Native)	Collision Energy (eV ^a^)
Diisoheptyl phthalate (DHpP)	MHHpP	d_4_-MHHpP	279.1/121.03	15
	MOHpP	d_4_-MOHpP	277.1/121.03	16
	MCHxP	d_4_-MCHxP	293.1/145.09	16

^a^ Collision energy applied in QExactive high-resolution mass spectrometer in parallel reaction mode.

**Table 2:** Selected percentiles of urinary concentrations (95% CI) of three oxidative metabolites (ng/mL) of diisoheptyl phthalate in a convenience sample of U.S. adults.

Urinary Metabolite	Collection Year	N	Percentile				Geometric Mean, ng/mL	Frequency of Detection (%)
			25th	50th	75th	90th		
MHHpP	2018–2019	205	0.25 (0.19, 0.29)	0.55 (0.4, 0.72)	1.71 (1.31, 2.2)	4.88 (3.39, 7.07)	0.59 (0.5, 0.7)	100
	2000	144	0.16 (0.13, 0.21)	0.44 (0.34, 0.57)	1.04 (0.87, 1.53)	1.99 (1.7, 2.4)	0.38 (0.31, 0.46)	96
MOHpP	2018–2019	205	<LOD ^a^	0.02 (<LOD, 0.04)	0.1 (0.07, 0.13)	0.23 (0.19, 0.3)	NA ^b^	57
	2000	144	0.07 (0.04, 0.09)	0.24 (0.17, 0.32)	0.64 (0.51, 0.99)	1.29 (1.04, 1.54)	0.19 (0.16, 0.24)	92
MCHxP	2018–2019	205	0.62 (0.49, 0.72)	1.3 (1.08, 1.6)	2.7 (2.26, 3.49)	5.16 (4.0, 7.66)	1.31 (1.15, 1.5)	100
	2000	144	0.93 (0.63, 1.41)	2.63 (1.96, 3.16)	5.11 (3.97, 6.27)	8.1 (7.17, 13.97)	2.01 (1.66, 2.43)	100

^a^ LOD—limit of detection; LOD—0.013 ng/mL for all three metabolites; ^b^ NA: Not calculated because detection frequency was <60%.
